# One-Year Outcomes of Preserflo^TM^ MicroShunt Implantation versus Trabeculectomy for Pseudoexfoliation Glaucoma

**DOI:** 10.3390/jcm12083000

**Published:** 2023-04-20

**Authors:** Matthias Nobl, Clara Grün, Stefan Kassumeh, Siegfried Priglinger, Marc J. Mackert

**Affiliations:** Department of Ophthalmology, Ludwig-Maximilians-University Munich, Mathildenstrasse 8, 80336 Munich, Germany

**Keywords:** pseudoexfoliation glaucoma, surgical treatment, trabeculectomy, Preserflo MicroShunt, minimally invasive glaucoma surgery, mitomycin C

## Abstract

This retrospective, single-center study evaluates the safety and efficacy of Preserflo^TM^ MicroShunt (MicroShunt) implantations compared to trabeculectomies (TETs) in patients diagnosed with pseudoexfoliation glaucoma (PEXG). A total of 31 eyes from 28 patients received a MicroShunt implantation, and 29 eyes from 26 patients received a TET. Surgical success was defined as an intraocular pressure (IOP) between 5 mmHg and 17 mmHg at the end of the follow-up period, no need for surgical revisions or secondary glaucoma surgery, and no loss of light perception. In the MicroShunt group, the mean IOP dropped from 20.8 ± 5.9 mmHg at baseline to 12.4 ± 2.8 mmHg (*p* < 0.0001) after one year. In the TET group, the mean IOP dropped from 22.3 ± 6.5 mmHg to 11.1 ± 3.7 mmHg (*p* < 0.0001) after 12 months. In both of the groups, the mean number of medications was reduced significantly (MicroShunt from 2.7 ± 1.2 to 0.2 ± 0.7; *p* < 0.0001 vs. TET from 2.9 ± 1.2 to 0.3 ± 0.9; *p* < 0.0001). Considering the success rates, 83.9% of the MicroShunt eyes achieved complete success, and 90.3% qualified for success at the end of the follow-up period. In the TET group, the rates were 82.8% and 93.1%, respectively. The postoperative complications were comparable between both groups. In conclusion, the MicroShunt implantation demonstrated non-inferiority regarding its efficacy and safety profile compared to TET in PEXG at a follow-up of one year.

## 1. Introduction

Pseudoexfoliation glaucoma (PEXG) is the most common identifiable cause of open-angle glaucoma (OAG) and accounts for about 20–25% of all OAGs [1]. Deposits of extracellular, fibrillary material from this systemic disease in the anterior segment of the eye and especially in the trabecular meshwork are the reason for the rise in intraocular pressure (IOP) [2]. Its appearance can be clinically asymmetric, but it affects the contralateral eye in 38% of cases within 10 years. The conversion to glaucoma in PEX is 32% after 10 years [3]. PEXG has a higher IOP than primary open-angle glaucoma (POAG) at presentation and a faster progression and is, therefore, the more aggressive disease [4,5]. Trabeculectomy (TET) is more often required in PEXG than in POAG, and, still, the long-term outcome is worse [6]. Although TET has been steadily improved over the last decades and remains globally accepted as the therapeutic gold standard, great efforts are being made to develop alternative surgical options. This is mainly due to the high rate of complications after TET, including hypotony with choroidal detachment, a flat anterior chamber, or hyphema, as well as a complex postoperative follow-up. Nowadays, minimally invasive glaucoma surgeries (MIGSs) are in vogue because they are safe and effective procedures with a short surgery time. The Preserflo^TM^ MicroShunt (MicroShunt; Santen, Osaka, Japan) is an ab-externo implant that drains the aqueous humor from the anterior chamber under the conjunctiva and was recently proven to be safe and effective in lowering the IOP in PEXG over one year [7,8]. Although MicroShunt implantation is not officially considered a MIGS procedure, it is less invasive and has a shorter surgical time compared to TET. The design of the implant with a balanced ratio between a length of 8.5 mm and an inner diameter of 70 µm is based on the Hagen–Poiseuille equation and, therefore, helps to avoid occlusion and hypotony [9]. It is made from soft, flexible, and highly biocompatible poly(styrene-block-isobutylene-block-styrene) materials. A pair of fins fixates the implant in the scleral pocket, maintaining proper alignment and preventing leakage alongside the device.

The aim of this study was to compare the effectiveness and safety of the gold standard TET with the MicroShunt in patients diagnosed with PEXG.

## 2. Methods

### 2.1. Study Design

This is an investigator-initiated, single-center, retrospective, interventional study. Patients with MicroShunt implantations augmented with mitomycin C (MMC) had surgery between July 2019 and December 2020. This group was compared to patients that received a TET with MMC from July 2016 to February 2019. The data were fully anonymized before the analysis. The study adhered to the tenets of the Declaration of Helsinki, and approval from the ethics committee (ID 22-0050) was obtained. Informed written consent was obtained from all patients prior to surgery.

### 2.2. Inclusion and Exclusion Criteria

All patients who had received a MicroShunt or a TET in the past were included, regardless of their lens status. The surgeries were performed because the patients had either an IOP above the target pressure or progression of the glaucoma disease under the maximum tolerable glaucoma medication. All patients who had received a MicroShunt surgery at the hospital since the introduction of the procedure in May 2019 were included. Patients who had received a TET since mid-2016 were included, which is when the electronic patient record was implemented. To be included in the study, patients had to be diagnosed with PEXG and be at least 18 years of age at the time of surgery. Prior surgery, especially prior incisional filtering glaucoma surgery, was not an exclusion criterion.

Patients with any other diagnosis than PEXG were not eligible for the study. A follow-up of less than eight months postoperatively was also an exclusion criterion.

### 2.3. Baseline Measurements

Baseline measurements were collected at the time of the indication for surgery. No washout of glaucoma medication was performed. Information about the glaucoma diagnosis, age, sex, lens status, laterality, central corneal thickness, IOP using Goldmann applanation tonometry, best-corrected visual acuity, number of IOP-lowering drugs, and history of prior surgery or laser treatment was recorded. In addition, standard automated perimetry was performed on a Humphrey Field Analyzer (Carl Zeiss AG, Oberkochen, Germany) before the patients underwent surgery.

### 2.4. Preoperative Management

The patients were told to perform preoperative preparation to achieve the best possible conjunctival condition. This included the discontinuation of all topical IOP-lowering drops in the eye that will be operated on 10 days before surgery. Instead, preservative-free dexamethasone eye drops were prescribed three times a day. To prevent an IOP increase, carbonic anhydrase inhibitors were administered orally three times daily.

### 2.5. Surgery

TET and the implantation of a MicroShunt were performed either under general anesthesia or retrobulbar anesthesia during an inpatient stay. The MicroShunt implantations were performed as described in detail by Nobl et al. [7]. The trabeculectomy procedure was similar to that described by Baker et al. [10]. However, we used three square sponges soaked with 0.2 mg/mL of MMC, which were inserted below the Tenon’s layer for 2 min after the preparation of the scleral flap. A sclerostomy was performed using a Kelly punch. The scleral flap was sutured with three non-releasable, single-knot 10-0 nylon sutures. Watertight closure of the conjunctiva was achieved with single-knot 10-0 vicryl sutures.

Both procedures were followed by a peribulbar injection of 4 mg of dexamethasone and the instillation of a dexa-gentamicin eye ointment, as well as an atropine eye ointment in the case of TET.

### 2.6. Postoperative Management

The postoperative therapy regime included the application of antibiotic eye drops four times per day for one week, the hourly application of steroid-containing eye drops for one week, which were tapered off over approximately two months, and the application of a steroid-containing eye ointment at night for four weeks.

During the inpatient stay of two to five days, the patients were examined daily. The first outpatient follow-up appointment was scheduled approximately one week after the inpatient discharge. The further follow-up visits were not performed in a standardized manner but were instead scheduled by the attending ophthalmic surgeon. Retrospectively, time periods of 1–4 weeks, 4–8 weeks, 3–4 months, 4–6 months, and 8–14 months after surgery were defined, and the data were analyzed accordingly.

The postoperative modulating procedures, such as injections of 5-fluoruracil (5-FU), laser suturolysis, anterior chamber revision with viscoelastics, or needling with 5-FU, as well as surgical revisions or restarts with local IOP-lowering therapy, were performed according to the decision of the treating ophthalmologist. The needling procedures were always performed in the operating room under local anesthesia using an operating microscope and a 30 G needle.

### 2.7. Outcome Measurements

The primary outcome was the percentage of eyes achieving complete success after 12 months of follow-ups. Failure was defined as an IOP of more than 17 mmHg or less than 5 mmHg at 12 months postoperatively, surgical revision, secondary glaucoma surgery, or loss of light perception. Needling was considered a postoperative supportive procedure and, therefore, not a criterion for failure. In contrast, surgical revision with opening of the conjunctiva and excision of scar tissue was considered a repeat glaucoma surgery and, thus, a treatment failure.

The absence of all failure criteria after one year was considered a complete success. If this was only achieved with the application of IOP-lowering drug therapy, it was considered a qualified success.

In addition to the primary IOP thresholds of 5 mmHg and 17 mmHg, further complete and qualified success rates were calculated for the different IOP thresholds. These were defined as IOP values between 5 mmHg and 15 mmHg and 5 mmHg and 19 mmHg.

### 2.8. Statistical Analysis

The means and standard deviations were calculated for the data presentation, unless otherwise noted. Percentages were given for the categorical values. The values for visual acuity were converted from Snellen to LogMAR. GraphPad Prism 9 (GraphPad Software, San Diego, CA, USA) was used to analyze the data and create the graphs. To test the two groups for significant differences, the Fisher’s exact test was used for the ordinal scaled data, and the non-parametric Mann–Whitney U test was used for the interval scaled data because the data were not normally distributed. For a comparison of more than two groups, a repeated-measures ANOVA with a Geisser–Greenhouse correction was used. A *p* value of < 0.05 was considered statistically significant.

## 3. Results

### 3.1. Study Population

A total of 31 eyes from 28 patients with MicroShunt implantations and 29 eyes from 26 patients with TETs were examined in this study. The baseline data of the study groups are shown in Table 1. There were significant differences regarding the age (TET 71.3 ± 8.3 years vs. MicroShunt 77.3 ± 7.7 years; *p* = 0.001) and the mean deviations in the visual field testing (*p* = 0.04). The IOP at baseline was comparable in both groups (TET 22.3 ± 6.5 mmHg vs. MicroShunt 20.8 ± 5.9 mmHg; *p* = 0.51). The percentage of pseudophakic eyes was 83.3% (26 eyes) in the MicroShunt group and 62.1% (18 eyes) in the TET group. Procedures combined with cataract surgery were performed in 10.3% (three eyes) of the eyes in the TET group and in 3.2% (one eye) of the eyes in the MicroShunt group. In the MicroShunt group, there were six eyes with previous glaucoma surgeries (two cyclophotocoagulation, two TET, one deep sclerectomy, and one iStent). In the TET group, there were three eyes (one deep sclerectomy, one XEN gel stent, and one iStent). The mean follow-up times were identical in both groups at 11 ± 2 months (*p* = 0.68).

### 3.2. Intraocular Pressure

**Figure 1:**
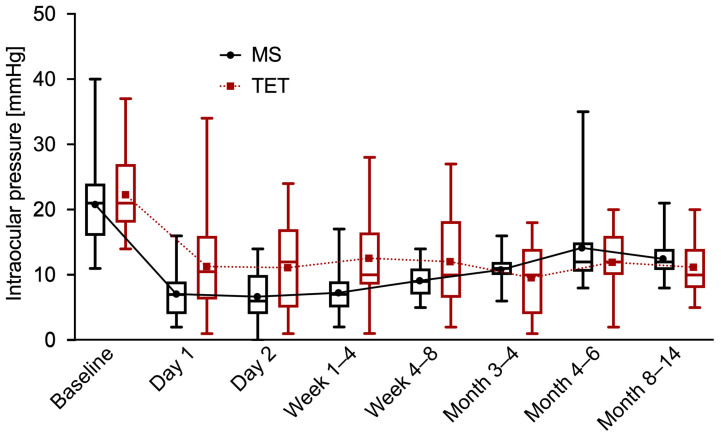
Intraocular pressure. Boxplots of intraocular pressure at each time of follow-up for MicroShunt implantation (MS) and trabeculectomy (TET). The mean values of intraocular pressure are connected by a dotted line.

**Figure 2:**
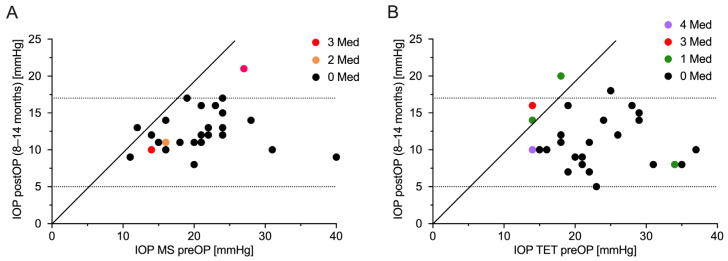
Scatter plot of intraocular pressure preoperatively versus one year postoperatively. (**A**) MicroShunt implantation and (**B**) trabeculectomy. The dotted lines represent the limits of surgical success, plotted according to the number of antiglaucomatous medications at one year postoperatively. IOP = intraocular pressure; MS = MicroShunt implantation; Med = antiglaucomatous medication; preOP = preoperative; postOP = postoperative; TET = trabeculectomy.

The IOP was significantly reduced from 20.8 ± 5.9 mmHg to 12.4 ± 2.8 mmHg (*p* < 0.0001) in the MicroShunt group after one year. In the TET group, the IOP was also significantly lowered from 22.3 ± 6.5 mmHg (*p* < 0.0001) to 11.1 ± 3.7 mmHg at the one-year follow-up. This corresponded to a reduction in the IOP of 40.4% in the MicroShunt group versus 50.2% in the TET group. The difference in the IOP reduction between the two groups was not statistically significant (*p* = 0.07). In general, there was a statistically significant difference in the IOP between the two groups only at 1–4 weeks (*p* = 0.009) after the surgeries. The development of the IOP over one year in both groups is shown in Figure 1. The scatterplot in Figure 2 illustrates the pre- and one-year postoperative IOP values in relation to the number of IOP-lowering drugs.

### 3.3. Medical Therapy

**Figure 3:**
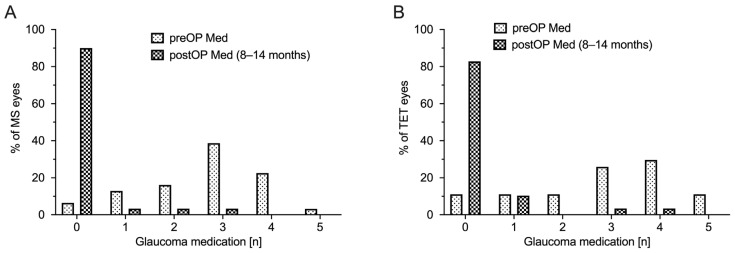
Number of antiglaucomatous medications. Number of antiglaucomatous medications preoperatively and one year postoperatively. (**A**) MS = MicroShunt implantation and (**B**) TET = trabeculectomy. PreOP = preoperative; postOP = postoperative.

**Figure 4:**
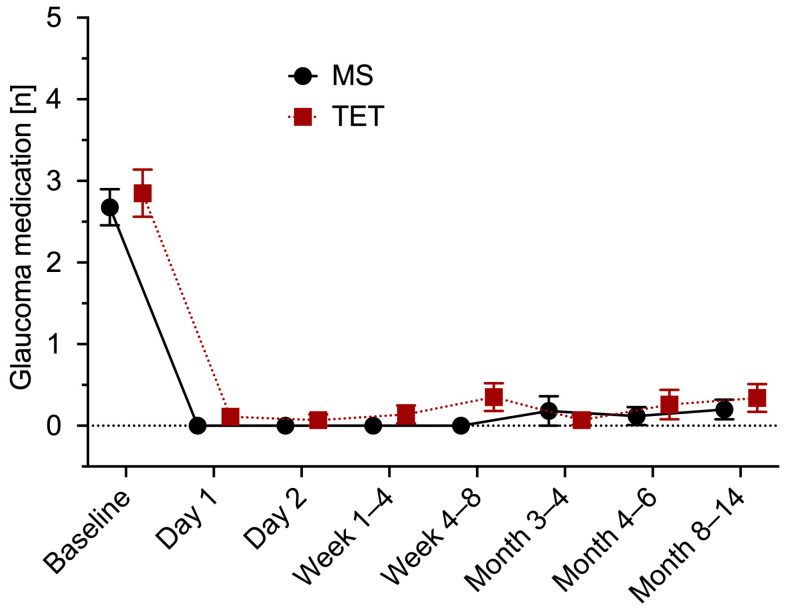
Medical therapy. Mean and standard error of the mean of the number of antiglaucomatous medications at each time of follow-up for MicroShunt implantation (MS) and trabeculectomy (TET).

The mean number of IOP-lowering drugs in the MicroShunt group was significantly reduced from 2.7 ± 1.2 to 0.2 ± 0.7 (*p* < 0.0001). There was also a statistically significant reduction in the TET group (from 2.9 ± 1.2 to 0.3 ± 0.9; *p* < 0.0001). No significant differences in the extent of reduction between the two groups were observed (*p* = 0.44). After one year, as shown in Figure 3, a total of eight eyes from seven patients (13.6%) had to be restarted with IOP-reducing medication to regulate the IOP (TET five eyes (17.2%) vs. MicroShunt three eyes (10%); *p* = 0.47). Figure 4 shows the changes in antiglaucomatous therapy over the course of the follow-ups.

### 3.4. Success Rates

The rates for complete success (MicroShunt 26/31 (83.9%) vs. TET 24/29 (82.8%); *p* > 0.9999) and qualified success (MicroShunt 28/31 (90.3%) vs. TET 27/29 (93.1%); *p* > 0.9999) were comparable in both groups. Likewise, the success rates calculated for the different IOP threshold values, shown in Table 2, were comparable between the two groups.

A total of four eyes required further surgical interventions (MicroShunt in three eyes (9.7%): two surgical revisions and one additional glaucoma surgery vs. TET in one eye (3.4%): one surgical revision). Cyclophotocoagulation was performed 4 months postoperatively in the case of one patient who underwent repeated glaucoma surgery. These four eyes were considered a treatment failure.

### 3.5. Postoperative Complications

Table 3 provides an overview of the postoperative complications in each group. The most common complications in both groups were hypotony (MicroShunt 14 eyes (45.2%) vs. TET 10 eyes (34.5%); *p* = 0.44) and choroidal detachment (MicroShunt 11 eyes (35.5%) vs. TET 5 eyes (17.2%); *p* = 0.15). A flat anterior chamber was seen postoperatively in five MicroShunt eyes and two TET eyes (16.1% vs. 6.9%; *p* = 0.43). While all of the cases of hypotony in the MicroShunt group appeared in the early postoperative phase and resolved within the first 4 weeks, hypotony in the TET group was also documented at later times. In addition, there was one case of prolonged hypotony, defined as lasting longer than 3 months, in the TET group. It appeared immediately after surgery and resolved on its own after 4 months without causing any complications or necessitating any intervention. Many cases of hypotony resolved on their own (MicroShunt six eyes vs. TET six eyes); some required conservative therapy with topical atropine and/or therapeutic contact lenses (MicroShunt five eyes vs. TET one eye), and others required a viscoelastic injection in the anterior chamber (MicroShunt two eyes vs. TET three eyes). Additionally, two of these three TET eyes needed transconjunctival scleral flap sutures.

In both groups, no case of blebitis, phthisis, or loss of light perception was documented. Furthermore, no cases of corneal decompensation were observed after one year of follow-up.

### 3.6. Postoperative Interventions

Postoperative subconjunctival injections of 5-FU were performed 60 times in total. In the MicroShunt group, 32 injections were performed (1.03 injections per eye; 11 eyes (35.5%) with only 1 injection; 11 eyes (35.5%) with 2 or more injections). In the TET group, 28 injections were counted (0.97 injections per eye; eight eyes (27.6%) with only 1 injection; nine eyes (31.0%) with 2 or more injections). There was no statistically significant difference in the number of eyes in both groups that required an injection of 5-FU (*p* = 0.42).

There was also no statistically significant difference when considering the number of needlings performed in both groups (*p* = 0.55). In the MicroShunt group, seven needlings were necessary (0.23 needlings per eye; five eyes (16.1%) with one needling; one eye (3.2%) with two needlings). The eyes in which the needling was performed showed an elevated IOP in four cases (57.1%), a flat bleb in five cases (71.4%), and Tenon cysts in 0 cases. In comparison, eight needlings were documented in the TET group (0.28 needlings per eye; eight eyes (27.6%) with one needling; zero eyes with two needlings). Needling was necessary in seven eyes (87.5%) because of an elevated IOP, in one eye (12.5%) because of a flat bleb, and in one eye (12.5%) because of a Tenon cyst.

## 4. Discussion

In this retrospective study of 60 eyes, the efficacy of reducing the IOP and antiglaucomatous medication and the safety of MicroShunt implantations augmented with MMC were compared to trabeculectomy, which is the gold standard for filtrating glaucoma surgeries. The long-term data suggest that TETs are effective in treating PEXG, even if the success rates are significantly lower than in POAG [11]. Currently, evidence of an IOP-lowering effect and the safety of the MicroShunt in PEXG is very limited. Recently, our study group published the effectiveness and safety of the MicroShunt in PEXG in comparison to POAG [7]. In the PEXG group, the mean IOP decreased from 21.4 ± 5.8 mmHg at baseline to 12.8 ± 3.0 mmHg (a reduction of 8.6 mmHg = 40.2%) after 12 months of follow-ups, and the number of antiglaucomatous medications dropped from 2.8 ± 1.3 at baseline to 0.3 ± 0.8. The current study showed a very similar decrease in the IOP for MicroShunt implantations from 20.8 ± 5.9 mmHg at baseline to 12.4 ± 2.8 mmHg (a reduction of 8.4 mmHg = 40.4%) and a reduction in antiglaucomatous medications from 2.7 ± 1.2 at baseline to 0.2 ± 0.7, which corroborate those findings.

To the best of our knowledge, there is no direct comparison between the MicroShunt implantation and the TET in PEXG by now. Recent studies comparing TET and MicroShunt implantations in POAG showed that both procedures are effective and safe in lowering the IOP [10,12,13,14]. In summary, these studies showed a significant IOP reduction for both surgical procedures from low twenties at baseline to low tens at the end of the follow-up period. The IOP reduction was greater in the TET group at the end of the follow-ups in all of the mentioned studies, reaching statistical significance in the studies of Baker et al. and Fili et al. [10,12]. Pillunat et al. compared the MicroShunt and TET in POAG in a non-randomized study that was part of the Dresden Glaucoma and Treatment Study (DGTS) over a period of 6 months, including 26 patients in each group [13]. At 6 months postoperatively, all patients were off their antiglaucomatous medication, except for one eye in the MicroShunt group. The authors concluded that both procedures were equally effective and safe in lowering the IOP in patients with POAG. Since the MicroShunt is less invasive, with a more convenient follow-up and fewer interventions needed postoperatively, they suggested that the MicroShunt might be recommended earlier in the treatment of glaucoma. Fili et al. compared the efficacy and safety of MicroShunt implantations and TETs in POAG in a large prospective study that included 150 eyes in each group, with a follow-up period of 12 months [12]. The number of topical medications administered 12 months after surgery was 0.4 ± 0.8 in the MicroShunt group and 0 in the TET group, compared to 2.5 ± 1.2 and 2.7 ± 0.9 at baseline (*p* = 0.00091 and *p* = 0.00072). In conclusion, MicroShunt implantation significantly reduced the IOP and the number of antiglaucomatous medications in POAG, but it was inferior to TET at reducing the IOP after a follow-up period of 12 months. Baker et al. presented one-year results from a prospective, randomized, multi-center study that included 395 patients undergoing MicroShunt implantation and 132 patients undergoing trabeculectomy [10]. The number of antiglaucomatous medications was significantly reduced in both groups (MicroShunt: from 3.1 ± 1.0 to 0.6 ± 1.1; *p* < 0.01; TET: from 3.0 ± 0.9 to 0.3 ± 0.9; *p* < 0.01). The authors concluded that the probability of success was lower in the MicroShunt group compared with the TET. Although reductions in the IOP and antiglaucomatous medications were observed in both groups, the TET group had a lower mean IOP on fewer medications. In the current study of patients with PEXG, the mean IOP reduction at one-year follow-up was statistically significant in both groups and reached a low target pressure of 12.4 mmHg in the MicroShunt group versus 11.1 mmHg in the TET group. Thus, the IOP values and the number of antiglaucomatous medications at the end of the follow-up period of the current study were similar to the results of the above-mentioned studies. Again, the IOP reduction in the TET group is greater than that in the MicroShunt group, although not statistically significant. Thus, both procedures are effective in lowering the IOP in PEXG with a low target pressure for advanced glaucoma. Therefore, our study proves the non-inferiority of the MicroShunt to the TET in surgically treating moderate-to-severe PEXG.

In addition, the IOP after one year in the MicroShunt group was similar to the IOP of different further studies evaluating the MicroShunt after one year [8,10,15,16,17,18]. Interestingly, in the period of 1 to 4 weeks postoperatively, the MicroShunt group had a significantly lower IOP in comparison to the TET group. The reason for this might be the fact that in TET, the IOP is higher immediately after surgery and adjusted in the early postoperative phase with laser suturolysis and needling with 5-FU. The reduction in the mean number of medications was equally significant in both groups, and only eight eyes required medication after one year. The complete success, which was defined as an IOP between 5 mmHg and 17 mmHg without medication or intervention after one year, was very high (MicroShunt 83.9% vs. TET 82.8%), with no significant differences between the two groups. Even at low target pressures in the range of 5 mmHg to 15 mmHg, the complete success rate was quite high (71.0% and 75.9%, respectively), with no significant differences. The success rates in our study are similar or even better compared to other studies evaluating the MicroShunt implant. A strength of our study and possibly also a reason for the good success rates could be the standardized preoperative preparation with the discontinuation of local IOP-lowering therapy, local application of preservative-free dexamethasone eye drops, and oral administration of a carbonic anhydrase inhibitor to achieve the best possible conjunctival situation. Schlenker et al. achieved a complete success rate of 76.9% after one year of follow-ups [18]. After 12 months of follow-ups, Scheres et al. reported 58% of complete and 79% of qualified success [17]. Durr et al. conducted a study on refractory glaucoma and found a complete success rate of 61% and a qualified success rate of 79.9% one year postoperatively [19]. Although the comparison of success rates between the studies is often compromised by using different success criteria, it gives a good impression of how to interpret the present results.

In the study, the population of patients in the TET group was younger than that of the MicroShunt group. This might reflect the real-world setting when considering which surgery is best for our patients. The TET is more invasive, and the aftercare is more complex; thus, there is a trend in performing MicroShunt surgeries in older patients. Furthermore, implant-free surgery is preferable for younger patients to avoid possible endothelial cell damage and, therefore, corneal decompensation in the long term [20,21]. More importantly, the baseline IOPs in both groups were identical. Regarding PEXG severity based on the mean deviation in the visual field, both groups were moderate-to-severe, but significantly worse in the TET group. It seems that for advanced glaucoma, TET has been chosen as the surgical modality to achieve a low target pressure.

Regarding surgical complications, we found no statistically significant differences among the two groups. In the early postoperative phase, although not statistically significant, more cases of hypotony and choroidal detachment were documented in the MicroShunt group. All of these resolved within four weeks after surgery. The rates of hypotony and choroidal detachments in the MicroShunt group were higher than the ones previously described in other studies on patients with POAG undergoing MicroShunt implantations [10,12]. In our previous study, we already noted that patients with PEXG have a higher risk of hypotony [7]. Whether the older age in the MicroShunt group or the PEXG-related tissue changes contribute to this difference remains unclear [22]. A possible explanation could be the altered tissue stiffness of the sclera in the context of PEXG, which, consequently, does not seal the entry canal to the same extent as in POAG, and, thus, a peritubular leakage is favored [23]. Maybe it would be beneficial to dilate the pupil at the end of surgery with atropine as we do in patients with trabeculectomy to prevent hypotony. Another option to prevent hypotony after MicroShunt implantations is the ab-externo intraluminal occlusion of the implant with a 9.0 or 10.0 nylon monofilament suture. After 3–4 weeks, the bleb is organized, and the suture can then be removed on a slit lamp, such as in Baerveldt glaucoma implants. Anterior chamber bleeding, and therefore, postoperative hyphema, may occur during the implantation of a MicroShunt, either during the placement of the scleral stitch channel or during the insertion of the implant. While hyphema was documented more often in the MicroShunt group, there were no statistically significant differences in comparison to the TET group. Corneal complications, such as corneal erosion or corneal edema, were comparable in both groups. Further studies with a longer follow-up are necessary to investigate whether the MicroShunt in the anterior chamber can lead to a disturbance of the endothelial function and, thus, to corneal complications later in the postoperative course. A migration of the MicroShunt through the conjunctiva was not observed in our study. During surgery, it is mandatory to cover the implant not only with the conjunctiva but also with the Tenon’s capsule to prevent the risk of implant extrusion.

Looking at postoperative interventions, such as injection of 5-FU or needling procedures, there was no significant difference in the amount of these procedures between the two groups. In addition to that, laser suturolysis was necessary in ten cases of the TET group (34.5%) in the early postoperative period. Taking this into account, the surgical aftercare in TET is more complex.

Due to its design, the study has certain limitations. First, due to retrospective data collection, active matching of the participants has not been possible. This resulted in significant baseline differences between the two groups. However, our study population represents an everyday patient population and, thus, allows for good conclusions to be drawn about everyday practice. Compared to other studies, the number of cases in our study is smaller, but it focused on a specific type of glaucoma. Finally, the follow-up is limited to one year. Further studies are necessary to draw conclusions regarding efficacy and safety in the long term.

## 5. Conclusions

Both surgical procedures are effective and safe for treating moderate-to-advanced PEXG with a low target pressure. The advantage of the MicroShunt is that it is a less invasive procedure with a faster surgery, quicker recovery, and more convenient aftercare, especially for older patients. The future will tell how we can address early postoperative hypotony after MicroShunt implantations in PEXG.

## Figures and Tables

**Table 1:** Demographic and clinical data.

	MicroShunt (n = 31)	Trabeculectomy (n = 29)	*p*
Age (y, mean ± SD)	77.3 ± 7.7	71.3 ± 8.3	0.001
Female [n (%)]	22 (71)	12 (41)	0.04
Pseudophakic [n (%)]	26 (84)	18 (62)	-
Bilateral cases [n (%)]	3 (10)	3 (10)	-
Combined surgery [n (%)]	1 (3)	3 (10)	-
CCT (µm, mean ± SD)	543 ± 45	542 ± 54	0.88
Baseline IOP (mmHg)	20.8 ± 5.9	22.3 ± 6.5	0.51
Baseline MD (mean ± SD)	−8.7 ± 6.8	−13.6 ± 8.7	0.04
Baseline BCVA (logmar, mean ± SD)	0.69 ± 0.25	0.55 ± 0.32	0.06
Baseline Medications (n, mean ± SD)	2.7 ± 1.2	2.9 ± 1.2	0.68

BCVA = best corrected visual acuity; CCT = central corneal thickness; IOP = intraocular pressure; MD = mean deviation; n = number; *p* = *p*-value; SD = standard deviation; y = years.

**Table 2:** Surgical success rates.

	MicroShunt (n = 31)	Trabeculectomy (n = 29)	*p*
complete success			
5–15 mmHg [% (n)]	71.0 (22/31)	75.9 (22/29)	0.77
5–17 mmHg [% (n)]	83.9 (26/31)	82.8 (24/29)	>0.9999
5–19 mmHg [% (n)]	83.9 (26/31)	82.8 (24/29)	>0.9999
qualified success			
5–15 mmHg [% (n)]	77.4 (24/31)	82.8 (24/29)	0.75
5–17 mmHg [% (n)]	90.3 (28/31)	93.1 (27/29)	>0.9999
5–19 mmHg [% (n)]	90.3 (28/31)	96.6 (28/29)	0.61

n = number; *p* = *p*-value.

**Table 3:** Postoperative complications.

	MicroShunt (n = 31)	Trabeculectomy (n = 29)	*p*
Hypotony [n (%)]	14 (45)	10 (35)	0.44
Choroidal detachment [n (%)]	11 (36)	5 (17)	0.15
Flat anterior chamber [n (%)]	5 (16)	2 (7)	0.43
Macular folds [n (%)]	2 (7)	1 (3)	>0.9999
Hyphema [n (%)]	5 (16)	2 (7)	0.43
Corneal complications [n (%)]	5 (16)	6 (21)	0.75
Corneal dellen [n (%)]	0 (0.0)	0 (0.0)	>0.9999
Corneal erosion [n (%)]	4 (13)	2 (7)	0.67
Corneal edema [n (%)]	1 (3)	4 (14)	0.19
Seidel positive [n (%)]	3 (10)	0 (0)	0.24
Implantat extrusion [n (%)]	0 (0)	n.a.	-
Blebitis [n (%)]	0 (0.0)	0 (0.0)	>0.9999
Loss of light perception [n (%)]	0 (0.0)	0 (0.0)	>0.9999

n = number; *p* = *p*-value; n.a. = not applicable.

## Data Availability

All relevant data were provided in the manuscript.
